# Lymph node metastasis and the physicochemical micro-environment of pancreatic ductal adenocarcinoma xenografts

**DOI:** 10.18632/oncotarget.18231

**Published:** 2017-05-26

**Authors:** Lise Mari K. Andersen, Catherine S. Wegner, Trude G. Simonsen, Ruixia Huang, Jon-Vidar Gaustad, Anette Hauge, Kanthi Galappathi, Einar K. Rofstad

**Affiliations:** ^1^ Group of Radiation Biology and Tumor Physiology, Department of Radiation Biology, Institute for Cancer Research, Oslo University Hospital, Oslo, Norway

**Keywords:** pancreatic carcinoma, metastasis, angiogenesis, hypoxia, interstitial fluid pressure

## Abstract

Pancreatic ductal adenocarcinoma (PDAC) patients develop lymph node metastases early and have a particularly poor prognosis. The poor prognosis has been shown to be associated with the physicochemical microenvironment of the tumor tissue, which is characterized by desmoplasia, abnormal microvasculature, extensive hypoxia, and highly elevated interstitial fluid pressure (IFP). In this study, we searched for associations between lymph node metastasis and features of the physicochemical microenvironment in an attempt to identify mechanisms leading to metastatic dissemination and growth. BxPC-3 and Capan-2 PDAC xenografts were used as preclinical models of human PDAC. In both models, lymph node metastasis was associated with high IFP rather than high fraction of hypoxic tissue or high microvascular density. Seven angiogenesis-related genes associated with high IFP-associated lymph node metastasis were detected by quantitative PCR in each of the models, and these genes were all up-regulated in high IFP/highly metastatic tumors. Three genes were mutual for the BxPC-3 and Capan-2 models: transforming growth factor beta, angiogenin, and insulin-like growth factor 1. Further comprehensive studies are needed to determine whether there is a causal relationship between the up-regulation of these genes and high IFP and/or high propensity for lymph node metastasis in PDAC.

## INTRODUCTION

Pancreatic ductal adenocarcinoma (PDAC) is the fourth leading cause of cancer-related deaths in the United States with a 5-year survival rate of 5-7% [1, 2], and unlike many other types of cancer, the incidence of PDAC is increasing [3]. Because most PDACs are resistant to chemotherapy and radiation treatment [4], surgery is the only treatment modality that may result in cure [5]. However, only 10-20% of the patients are eligible for surgery at presentation, and even then, the outcome is poor with a 5-year survival rate of 15-20% [6, 7]. Metastatic spread into and through lymphatics and lymph nodes occurs frequently in PDAC patients, and the presence of lymph node metastases is strongly associated with poor prognosis [8]. Ultimately, most patients die from metastatic disease in the liver, lungs, or peritoneum [9].

The microenvironment of PDACs is characterized by an abundant desmoplastic stroma that may occupy up to 80% of the tumor volume [10–12]. The PDAC stroma consists of a dynamic assortment of extracellular matrix components including fibronectin, collagen, proteoglycans, and hyaluronic acid, nonmalignant cells including fibroblasts, endothelial cells, and immune cells, and soluble proteins such as growth factors and cytokines [12]. Recent investigations have revealed that the PDAC stroma represents a physical barrier to the delivery of chemotherapeutic agents and simultaneously supports tumor growth and promotes metastatic dissemination [10–12].

The development of an abundant stroma during tumor growth distorts the architecture of the normal pancreas, resulting in an abnormal configuration of blood vessels and lymphatics in PDACs [13, 14]. Histological examinations of tumors in genetically engineered mouse models of PDAC and human PDACs have revealed that PDACs are hypovascular [15, 16]. Thus, the microvascular density (MVD) has been measured to be significantly lower in PDACs than in the normal pancreas, and furthermore, it has been shown that PDACs have significantly fewer large-diameter blood vessels (diameter > 10 μm) than the adjacent non-tumorigenic pancreatic tissue [17–21]. The paucity of large-diameter vessels is believed to be a consequence of vessel compression induced by water retention in the hyaluronan component of the tumor stroma [21–24]. The geometric resistance to blood flow is high in microvascular networks showing high fractions of low-diameter vessels, resulting in elevated microvascular pressure and low erythrocyte velocities [25]. Tumors with low MVD and high fractions of low-diameter vessels may develop interstitial hypertension and hypoxic regions during growth [26, 27], and indeed, preclinical and clinical investigations have revealed that PDACs may show highly elevated interstitial fluid pressure (IFP) [21, 28, 29] as well as high fractions of hypoxic tissue [30–36].

The dense desmoplastic stroma has been suggested to be a determinant of the aggressive metastatic growth of PDACs [10–12]. There is some evidence that PDAC metastasis is promoted by direct interactions between the parenchymal tumor cells and the cellular and/or matrix components of the stroma [37, 38]. Because studies of a large number of cancer types have revealed that metastasis is associated with several parameters of the physicochemical microenvironment of the tumor tissue, including MVD [39, 40], IFP [41, 42], and extent of hypoxia [43–45], it is possible that also the metastatic propensity of PDACs is determined by these parameters. This possibility was investigated in the present work by using intramuscular BxPC-3 and Capan-2 PDAC xenografts as preclinical tumor models. Tumors were subjected to measurement of IFP when having grown to an appropriate size, and then the tumors were resected and prepared for immunohistochemical assessment of MVD and hypoxic fraction, and the host mice were examined for the presence of lymph node metastases.

## RESULTS

### Metastasis assay

**Figure 1:**
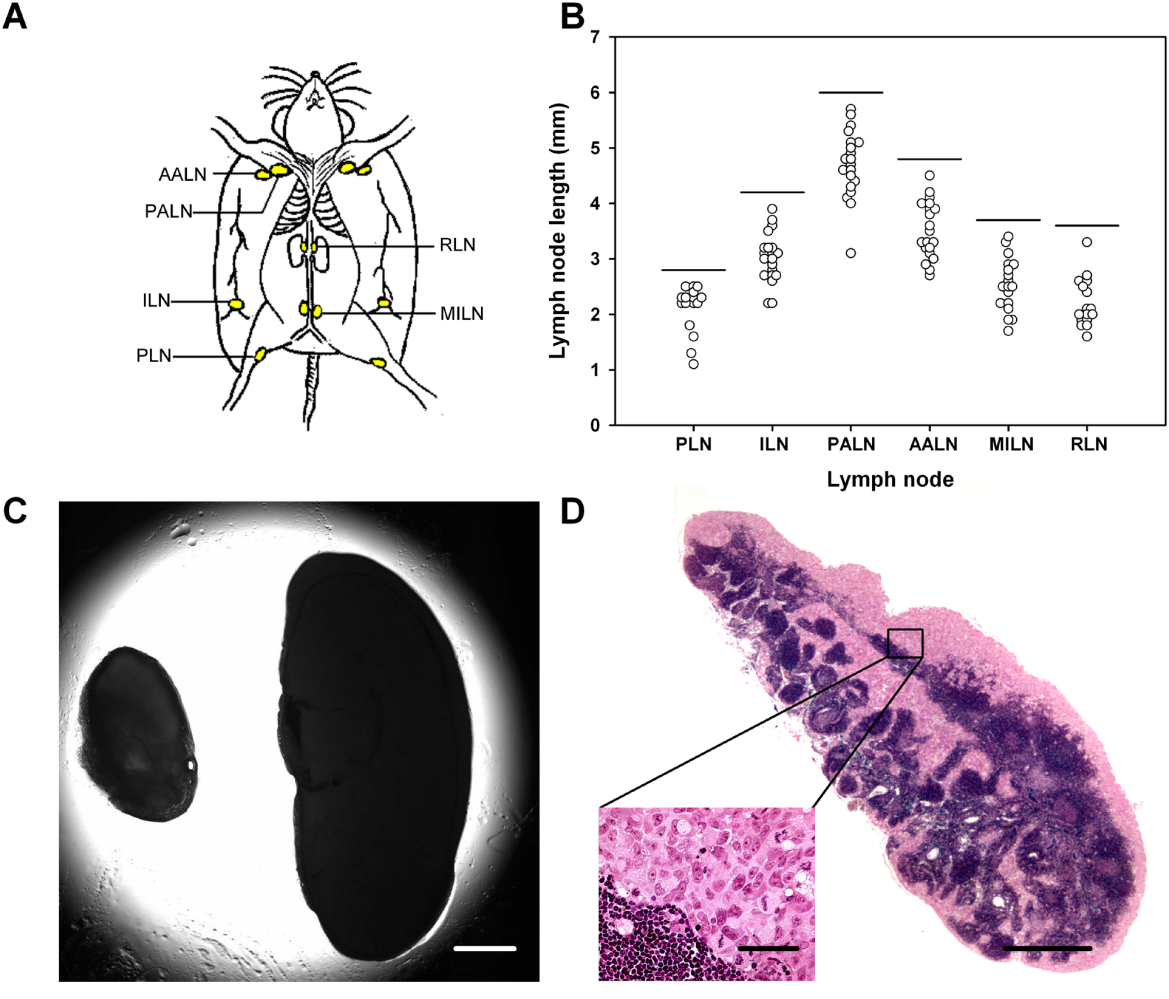
Metastasis assay **(A)** Six pairs of lymph nodes were examined for metastatic growth: PLN, popliteal lymph nodes; ILN, inguinal lymph nodes; PALN, proper axillary lymph nodes; AALN, accessory axillary lymph nodes; MILN, medial iliac lymph nodes; RLN, renal lymph nodes. **(B)** Length of these lymph nodes in mice without tumors. The horizontal lines indicate the threshold lymph node lengths, that is, lymph nodes with lengths above the threshold values were considered to show metastatic growth. The threshold values are 2.8 mm (PLN), 4.2 mm (ILN), 6.0 mm (PALN), 4.8 mm (AALN), 3.7 mm (MILN), and 3.6 mm (RLN). **(C)** Representative examples of lymph nodes without (left) and with (right) metastatic growth. Scale bar: 1.0 mm. **(D)** Histological section prepared from a representative lymph node with metastatic growth. Scale bars: 100 μm (high magnification) and 1.0 mm (low magnification).

Intramuscular tumors were initiated in the left hind leg, and the metastatic status of the host mice was assessed by examining the popliteal lymph nodes (PLN), inguinal lymph nodes (ILN), proper axillary lymph nodes (PALN), accessory axillary lymph nodes (AALN), medial iliac lymph nodes (MILN), and renal lymph nodes (RLN; Figure 1A). These lymph nodes differed in size in mice without tumors, and the size of a given lymph node differed among individual mice (Figure 1B). In tumor-bearing mice, lymph nodes with lengths above those indicated by the horizontal lines in Figure 1B were considered to be metastatic, that is, lymph nodes longer than 2.8 mm (PLN), 4.2 mm (ILN), 6.0 mm (PALN), 4.8 mm (AALN), 3.7 mm (MILN), and 3.6 mm (RLN). Histological examinations confirmed that lymph nodes with lengths above these threshold values always contained pancreatic tumor cells. In general, a metastatic lymph node was substantially larger than the corresponding non-metastatic lymph node (Figure 1C), reflecting that most metastatic lymph nodes were pervaded by malignant tissue (Figure 1D).

### Tumor histology

**Figure 2:**
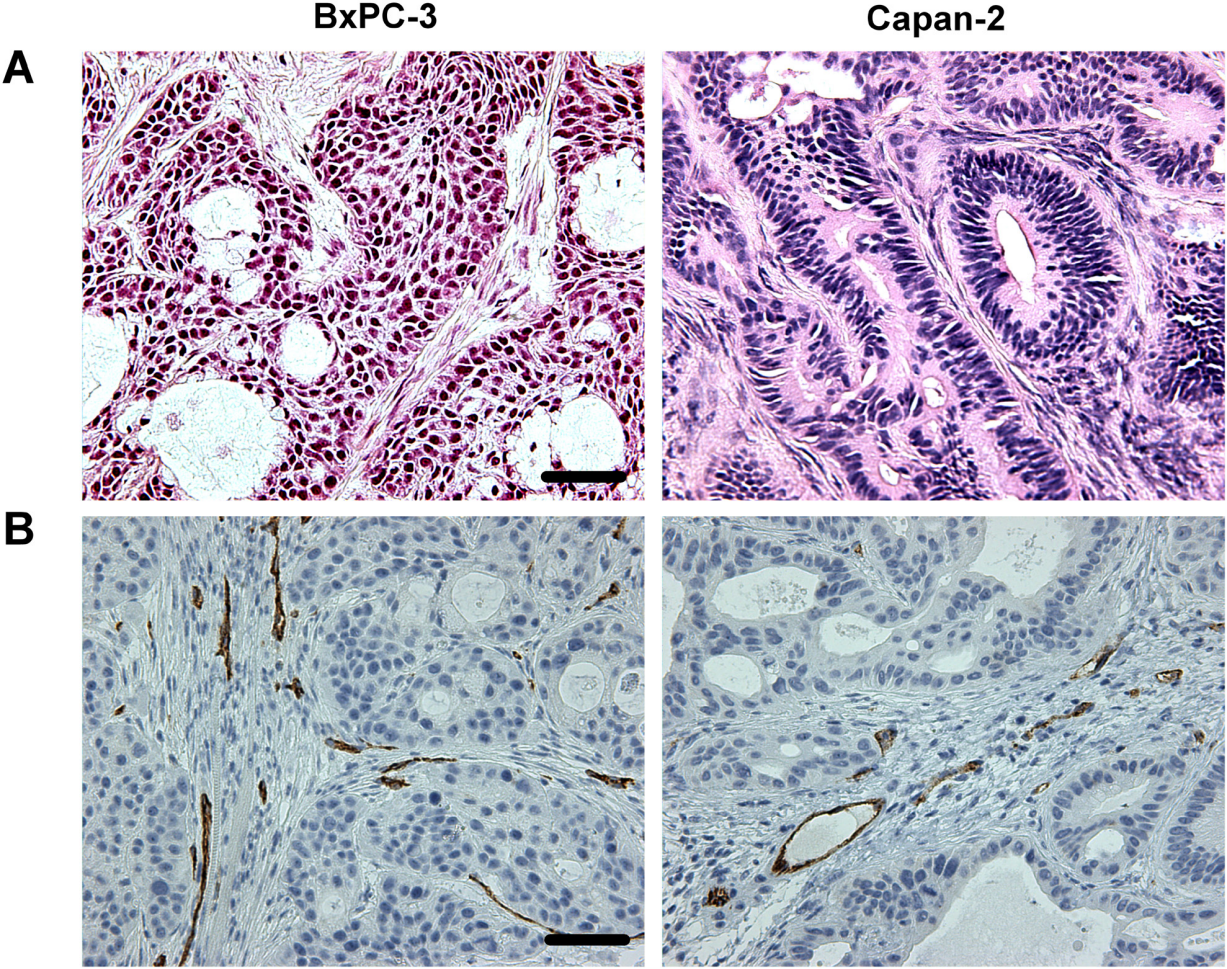
Histological appearance of BxPC-3 and Capan-2 PDAC xenografts **(A)** HE stained preparations. Scale bar: 500 μm. **(B)** Immunohistochemical preparations stained for blood vessels by using an antibody against the endothelial cell marker CD31. Scale bar: 500 μm.

**Figure 3:**
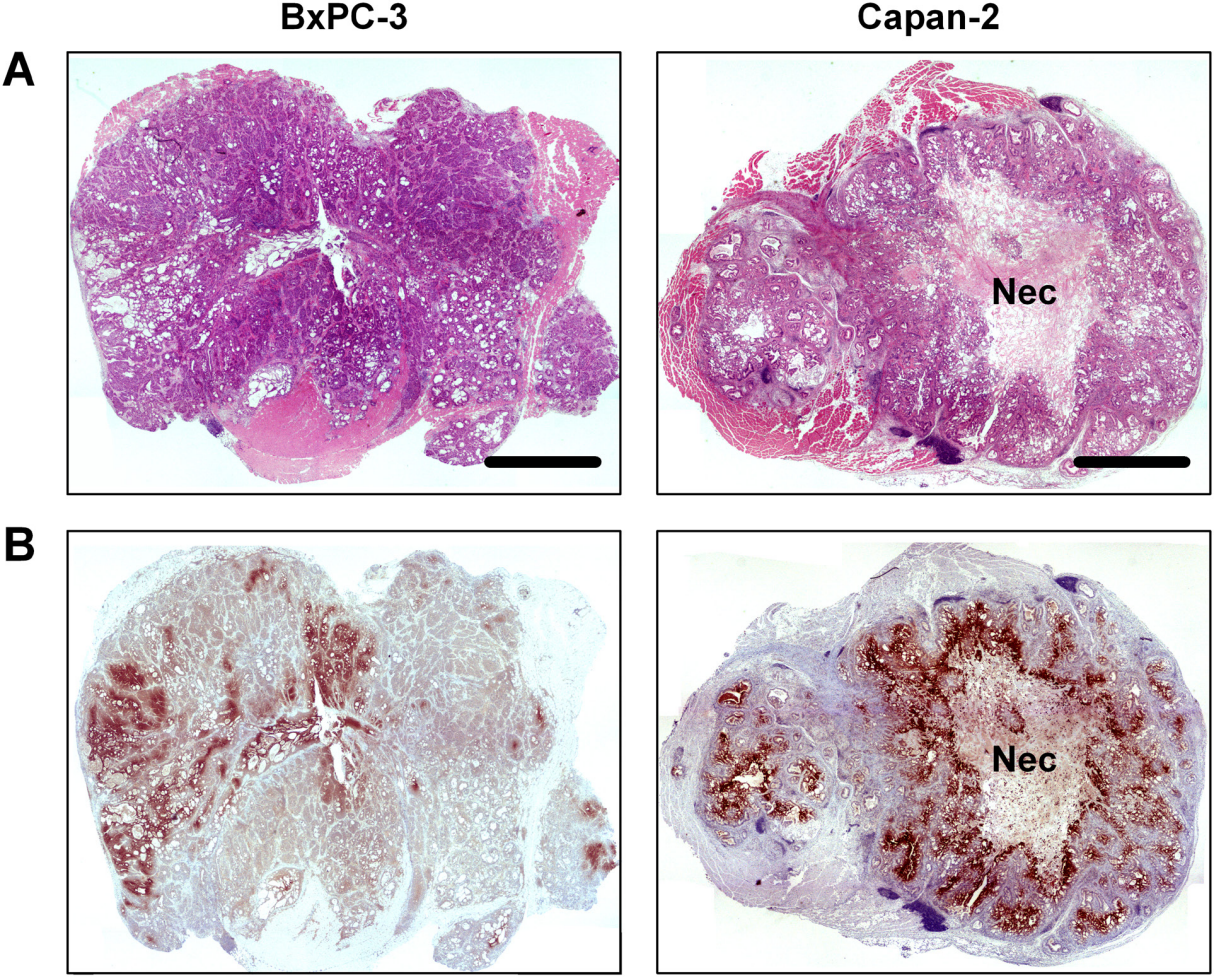
Whole mount histological preparations of BxPC-3 and Capan-2 PDAC xenografts **(A)** HE stained preparations. Scale bar: 2.0 mm. Nec: necrosis. **(B)** Immunohistochemical preparations stained for hypoxia by using an antibody against the hypoxia marker pimonidazole. Dark brown: hypoxic tissue.

The histological appearance of the tumor models is illustrated in Figure 2. Both models were differentiated and showed ductal structures; however, the level of differentiation was higher in Capan-2 tumors than in BxPC-3 tumors (Figure 2A). CD31 was used as a marker of endothelial cells, and histological preparations stained for CD31 showed that the blood vessels in both models were located primarily in the connective tissue rather than in the tumor parenchyma (Figure 2B). Pimonidazole was used as a marker of tumor hypoxia, and both models showed foci of pimonidazole-positive cells in morphologically intact tissue, and positive pimonidazole staining was always seen in tissue adjacent to necrotic and fibrotic tumor regions (Figure 3).

### Physicochemical tumor microenvironment and lymph node metastasis

**Figure 4:**
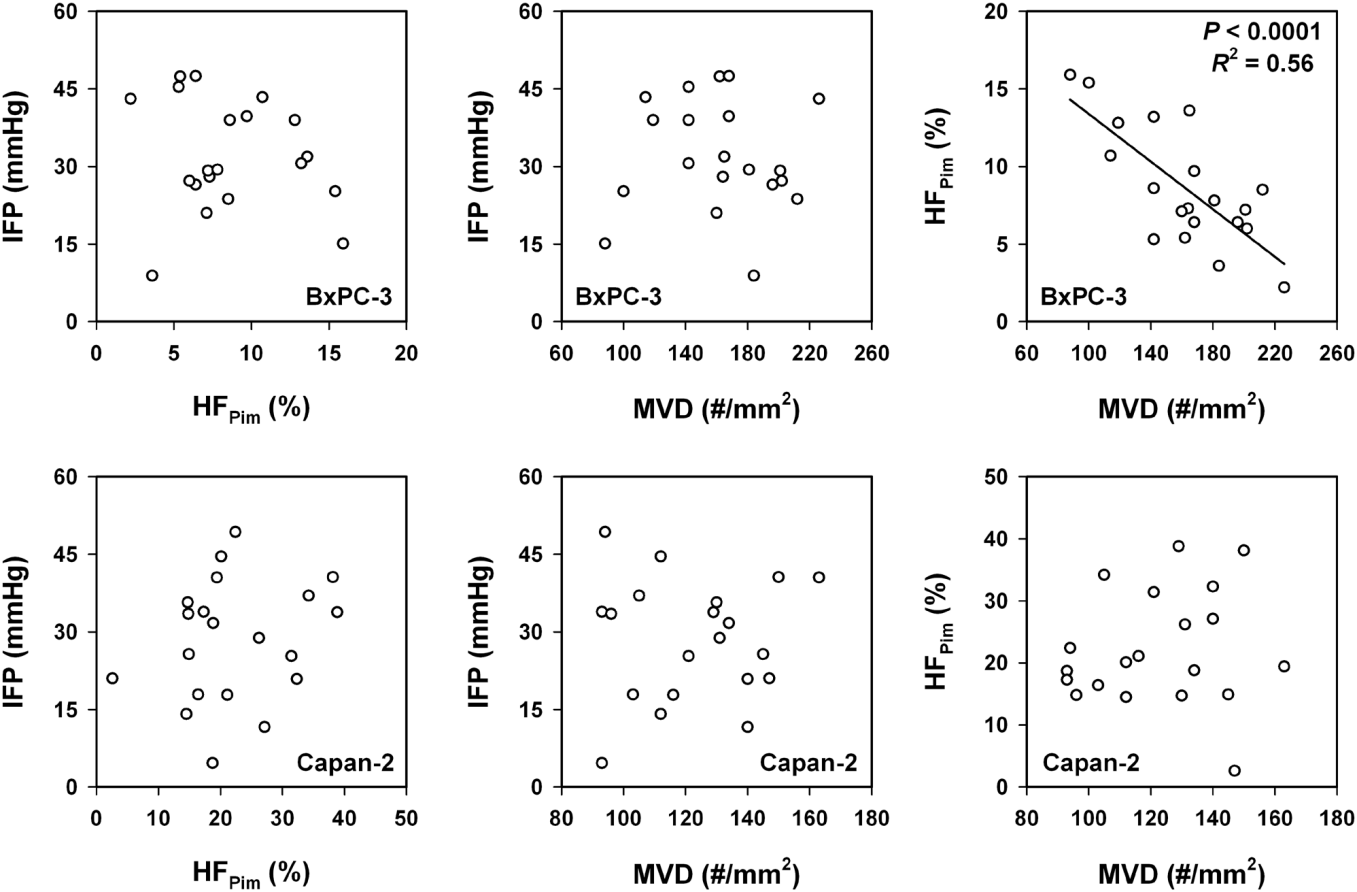
The physicochemical microenvironment of PDAC xenografts Plots of interstitial fluid pressure (IFP) *vs* hypoxic fraction (HF_Pim_), IFP *vs* microvascular density (MVD), and HF_Pim_ *vs* MVD for BxPC-3 and Capan-2 tumors. Symbols: individual tumors. Curve: linear regression line.

Associations between lymph node metastasis and the physicochemical microenvironment of tumors were searched for by measuring IFP, hypoxic fraction (HF_Pim_), and MVD in 20 tumors of each model and relating the measured values to the metastatic status of the host mice. The physicochemical microenvironment differed substantially among the individual tumors in both models (Figure 4). IFP ranged from 8.9 mmHg to 47.5 mmHg (BxPC-3) and from 4.6 mmHg to 49.3 mmHg (Capan-2), HF_Pim_ ranged from 2.2% to 15.9% (BxPC-3) and from 2.6% to 38.8% (Capan-2), and MVD (#/mm^2^) ranged from 88 to 226 (BxPC-3) and from 93 to 163 (Capan-2). IFP did not correlate with HF_Pim_ or MVD in any of the models, whereas HF_Pim_ decreased with increasing MVD in the BxPC-3 model (*P* < 0.0001; *R*^2^ = 0.56), but not in the Capan-2 model (*P* > 0.05).

**Figure 5:**
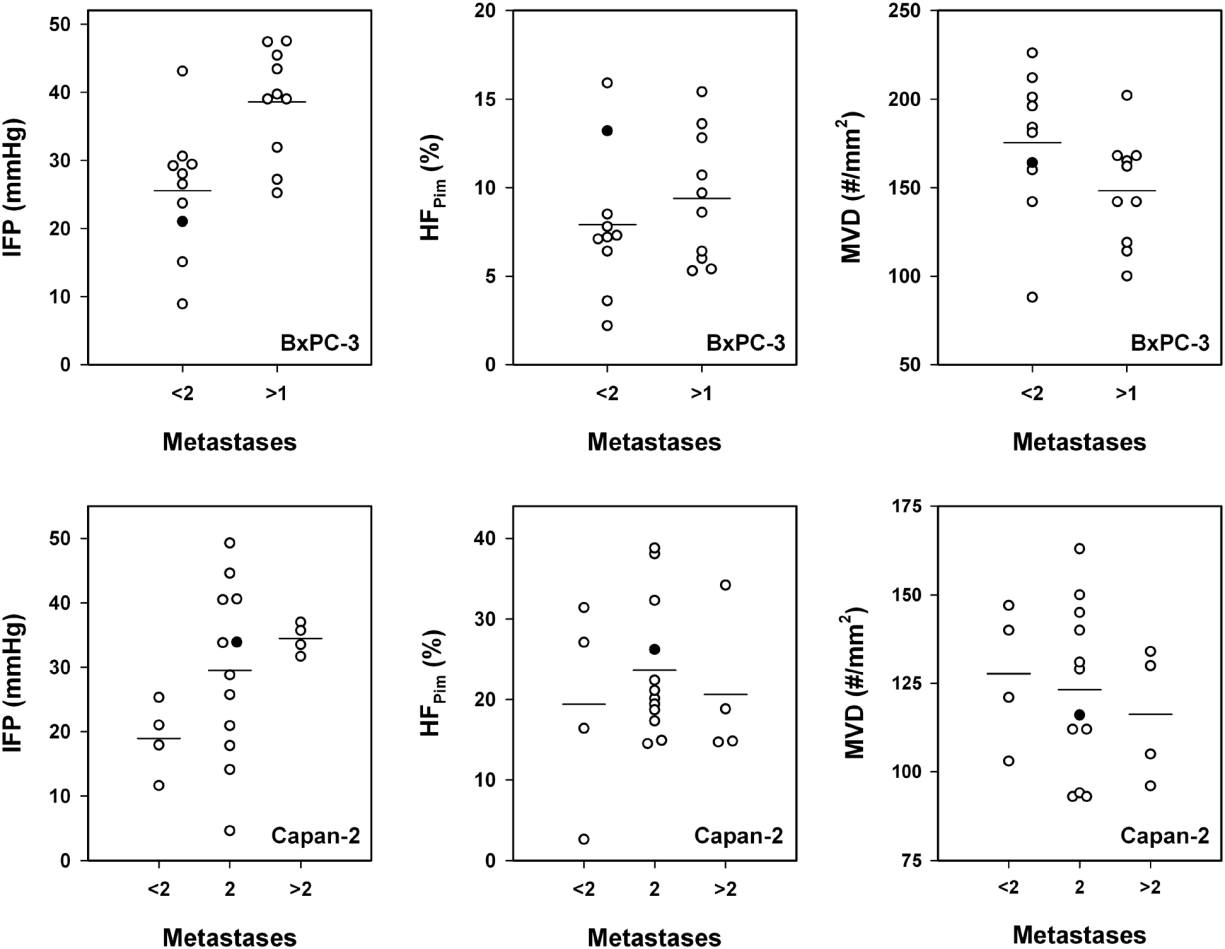
The metastatic propensity of PDAC xenografts Interstitial fluid pressure (IFP), hypoxic fraction (HF_Pim_), and microvascular density (MVD) in BxPC-3 tumors with low (*n* < 2) and high (*n* > 1) metastatic propensity and in Capan-2 tumors with low (*n* < 2), medium (*n* = 2), and high (*n* > 2) metastatic propensity. *n*: number of lymph nodes showing metastatic growth. Symbols: individual tumors. Closed symbols: tumors depicted in Figure 3. Horizontal lines: mean values.

Metastasis to the liver, lungs, or peritoneum was not detected in any of the 40 mice included in the study. Thirty-seven mice had developed metastatic growth in one or more lymph nodes, implying that only three mice (two with BxPC-3 tumors and one with a Capan-2 tumor) were metastasis-free. It is possible that also these three mice would have developed lymph node metastases if the primary tumor had been allowed to grow beyond a size of 600 mm^3^. Ten of the 20 mice with BxPC-3 tumors showed two or more metastasis-positive lymph nodes (*n* > 1) whereas the other 10 mice showed none or one lymph node with metastatic growth (*n* < 2; Figure 5). The more metastatic tumors had significantly higher IFP than the less metastatic tumors (*P* = 0.0018), whereas these two tumor groups did not differ significantly in HF_Pim_ (*P* > 0.05) or MVD (*P* > 0.05). Of the 20 mice with Capan-2 tumors, four mice showed none or one lymph node with metastatic growth (*n* < 2), 12 mice had developed two metastasis-positive lymph nodes (*n* = 2), and four mice showed three or more lymph nodes with metastases (*n* > 2; Figure 5). These three groups did not differ significantly in HF_Pim_ (*P* > 0.05) or MVD (*P* > 0.05). However, one-way ANOVA followed by the Bonferroni's test revealed that the IFP was significantly higher in the (*n* > 2)-group than in the (*n* < 2)-group (*P* = 0.012), whereas there was no significant difference in IFP between the (*n* = 2)-group and the (*n* < 2)-group (*P* > 0.05) or the (*n* > 2)-group (*P* > 0.05). Furthermore, the Student *t* test showed that the IFP of the 16 tumors giving rise to two or more than two lymph nodes with metastases was significantly higher than the IFP of the four tumors giving rise to none or one metastasis-positive lymph node (*P* = 0.035). Taken together, these data suggest that the metastatic propensity of the BxPC-3 and Capan-2 tumors was associated with high IFP, but not with high HF_Pim_ or MVD.

### Angiogenesis-related genes associated with interstitial hypertension-associated lymph node metastasis

**Figure 6:**
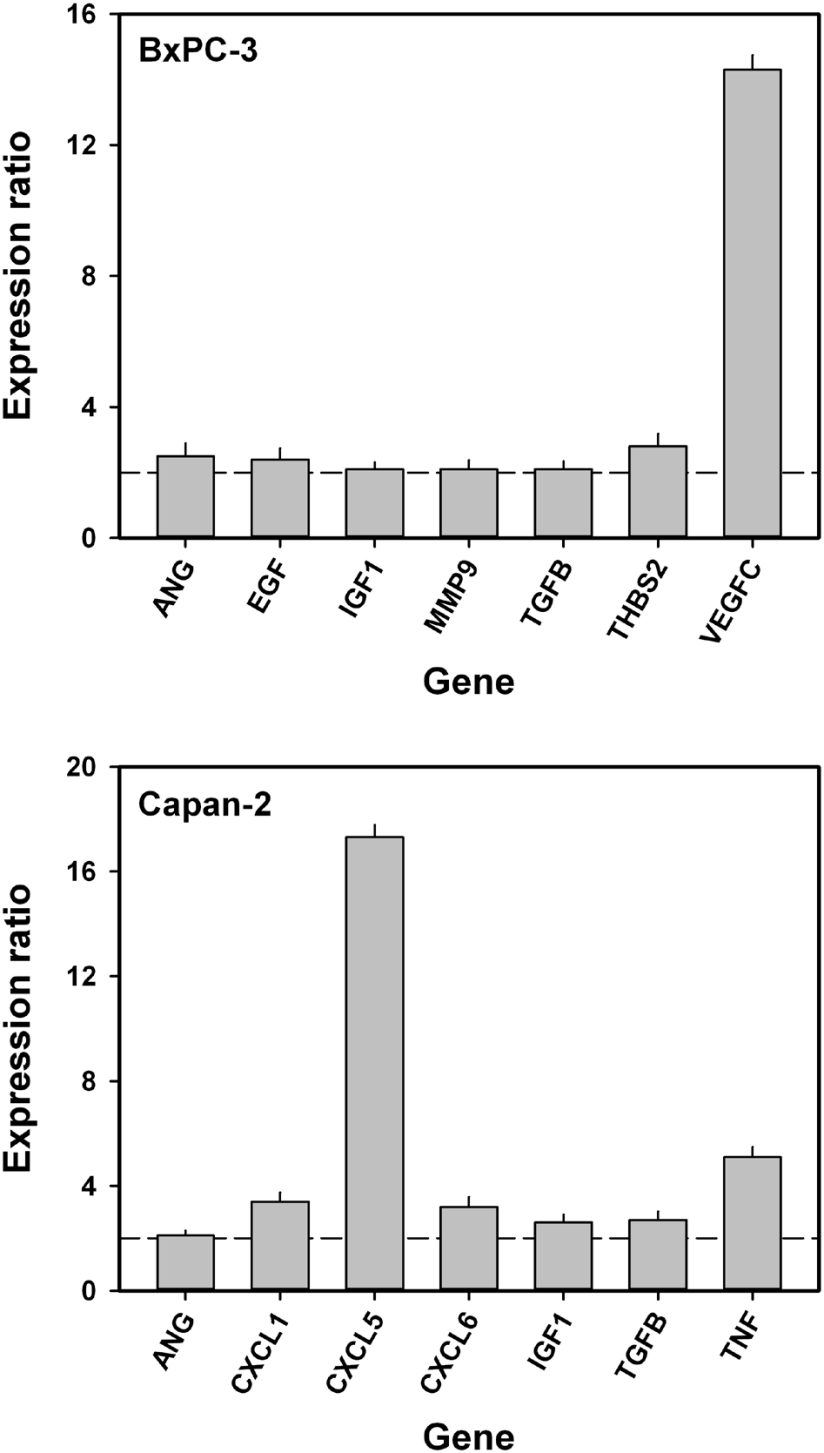
Angiogenesis-related genes associated with interstitial hypertension-associated lymph node metastasis in BxPC-3 and Capan-2 PDAC xenografts Mean expression level in high IFP/highly metastatic tumors divided by mean expression level in low IFP/poorly metastatic tumors. Dashed horizontal lines indicate an expression ratio of 2. The following genes were up-regulated in high IFP/highly metastatic BxPC-3 tumors: ANG, angiogenin (*P* = 0.023); EGF, epidermal growth factor (*P* = 0.030); IGF1, insulin-like growth factor 1 (*P* = 0.039); MMP9, matrix metallopeptidase 9 (*P* = 0.048); TGFB, transforming growth factor beta (*P* = 0.041); THBS2, thrombospondin 2 (*P* = 0.016); and VEGFC, vascular endothelial growth factor-C (*P* < 0.0001). The following genes were up-regulated in high IFP/highly metastatic Capan-2 tumors: ANG (*P* = 0.046); CXCL1, chemokine (C-X-C motif) ligand 1 (*P* = 0.010); CXCL5, chemokine (C-X-C motif) ligand 5 (*P* < 0.0001); CXCL6, chemokine (C-X-C motif) ligand 6 (*P* = 0.012); IGF1 (*P* = 0.027); TGFB (*P* = 0.021); and TNF, tumor necrosis factor (*P* = 0.0025). ANG, IGF1, and TGFB were up-regulated in high IFP/highly metastatic tumors in both BxPC-3 and Capan-2 tumors. Columns and bars: mean values ± SD (*N* = 4 tumors).

To identify genes associated with interstitial hypertension-associated lymph node metastasis, quantitative PCR was carried out on highly metastatic primary tumors (*n* = 2) with high IFP (IFP > 40 mmHg) and poorly metastatic primary tumors (*n* < 2) with low IFP (IFP < 25 mmHg) by using a PCR array for expression profiling of angiogenesis-related genes. Four tumors of each category were examined in both models. In the BxPC-3 model, we identified seven genes that were significantly up-regulated in the high IFP/highly metastatic tumors and showed a mean expression level in these tumors that was >2-fold higher than that in the low IFP/poorly metastatic tumors (Figure 6). Vascular endothelial growth factor-C (VEGF-C) was by far the angiogenesis-related gene that showed the largest difference in expression level between high IFP/highly metastatic tumors and low IFP/poorly metastatic tumors in the BxPC-3 model. By the same criteria, seven angiogenesis-related genes associated with interstitial hypertension-associated metastasis were also identified in the Capan-2 model (Figure 6). In this model, the largest differences in expression level between high IFP/highly metastatic tumors and low IFP/poorly metastatic tumors were seen for chemokine (C-X-C motif) ligand 5 (CXCL5) and tumor necrosis factor (TNF). The BxPC-3 and Capan-2 models had three angiogenesis-related genes in common that were associated with interstitial hypertension-associated metastasis: angiogenin (ANG), insulin-like growth factor-1 (IGF1), and transforming growth factor beta (TGFB).

### Tumor-associated lymphatics and lymph node metastasis

**Figure 7:**
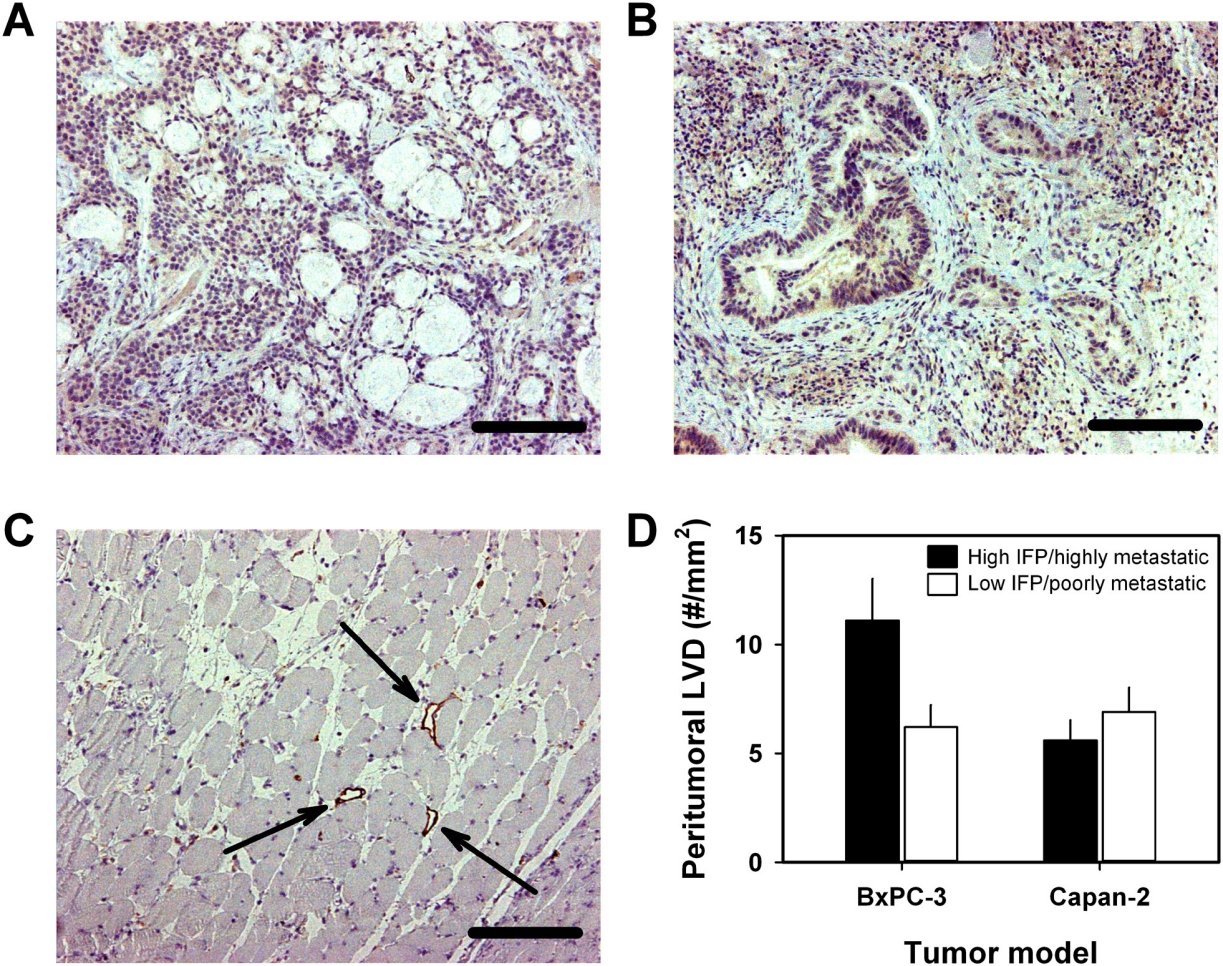
Lymphatics associated with BxPC-3 and Capan-2 PDAC xenografts Immunohistochemical preparations stained for lymphatics by using an antibody against the endothelial cell marker LYVE-1. **(A)** BxPC-3 tumor showing no intratumoral lymphatics. Scale bar: 400 μm. **(B)** Capan-2 tumor showing no intratumoral lymphatics. Scale bar: 400 μm. **(C)** Peritumoral muscle tissue showing lymphatics. Arrows point to large open lymphatics. Scale bar: 400 μm. **(D)** Peritumoral lymph vessel density (LVD) of high IFP/highly metastatic and low IFP/poorly metastatic BxPC-3 and Capan-2 tumors. Columns and bars: mean values ± SD (*N* = 4 tumors).

Lymph node metastasis in human PDAC has been shown to be associated with high expression of VEGF-C and high density of lymphatics [8], and because high IFP/highly metastatic BxPC-3 tumors showed highly elevated expression of VEGF-C, we examined the presence of tumor-associated lymphatics in high IFP/highly metastatic and low IFP/poorly metastatic tumors by using an immunohistochemical assay and LYVE-1 as a marker of lymphatic endothelial cells. Positive intratumoral staining for LYVE-1 could not be detected in either of the PDAC models (Figure 7A and 7B), whereas the peritumoral muscle tissue of both models showed LYVE-1-positive lymphatics (Figure 7C). In the BxPC-3 model, the density of peritumoral lymphatics was significantly higher in high IFP/highly metastatic tumors than in low IFP/poorly metastatic tumors (*P* = 0.0011), whereas in the Capan-2 model, high IFP/highly metastatic and low IFP/poorly metastatic tumors did not differ significantly in peritumoral lymph vessel density (Figure 7D), consistent with the VEGF-C expression data in Figure 6.

## DISCUSSION

Preclinical studies of PDAC are currently being carried out by using a wide variety of tumor models, including transgenic mouse models, ectopic or orthotopic patient-derived xenograft models, and cell line-derived xenograft models transplanted to subcutaneous or intramuscular sites. Transgenic models have the advantage that the tumors arise spontaneously in the pancreas of immune-competent mice, whereas xenograft models have the advantage that the tumors consist of human malignant cells. In this study, intramuscular BxPC-3 and Capan-2 PDAC xenografts were used as models of human PDAC, and similar to other preclinical PDAC models, these models have some significant limitations. The intramuscular site is an ectopic site for PDAC, and therefore, the tumor microenvironment of intramuscular PDAC xenografts cannot completely recapitulate the very complex desmoplastic tumor microenvironment of human PDACs [20, 28]. Lymph node metastasis of PDAC is promoted by direct interactions between the parenchymal tumor cells and the cellular and matrix components of the tumor stroma [37, 38], and consequently, the metastatic propensity of ectopic PDAC models may not necessarily be similar to that of PDACs in cancer patients. Furthermore, the incidence and the site of development of lymph node metastases depend on the density and structure of the peritumoral lymphatics [8, 13, 14], and the peritumoral lymphatic network of intramuscular PDAC xenografts may differ from that of human PDACs. Importantly, patients with PDAC primarily develop intra-abdominal lymph node metastases, and so did the intramuscular BxPC-3 and Capan-2 PDAC xenografts.

It is important to note that the histological appearance of the BxPC-3 and Capan-2 PDAC xenografts was similar to that of PDACs in humans. Both PDAC models showed distinct ductal structures enclosed by an abundant desmoplastic stroma. The stroma was seen as a dense matrix of connective tissue fibers appearing in thick filament bundles, and the majority of the blood vessels were located within these bundles. We have shown previously that the extracellular matrix of BxPC-3 and Capan-2 xenografts is rich in collagen I [46], consistent with the observation that collagen I is up-regulated in human PDAC [47].

There is significant evidence from studies of experimental tumors that tumor hypoxia, IFP, and other parameters of the physicochemical tumor microenvironment are strongly dependent on the site of tumor transplantation [26]. In this study, the physicochemical microenvironment differed considerably among the individual tumors of the BxPC-3 and Capan-2 PDAC models, and the numerical values of the microenvironmental parameters were similar to those reported for other preclinical models of PDAC and human PDACs. The MVD (#/mm^2^) ranged from 88 to 226 (BxPC-3) and from 93 to 163 (Capan-2), and these values are comparable to the range of 88-177 measured in an investigation of 52 PDAC patients [18]. Fraction of hypoxic tissue differed from 2.2% to 15.9% (BxPC-3) and from 2.6% to 38.8% (Capan-2), and these fractions are similar to those of 0-25% measured in a study of 16 orthotopic patient-derived PDAC xenograft models [36] and those of 0-26% measured in a study of 10 PDAC patients [33, 34]. IFP varied from 8.9 mmHg to 47.5 mmHg (BxPC-3) and from 4.6 mmHg to 49.3 mmHg (Capan-2), and these IFP values are within the same range as those measured in intramuscular tumor models of several cancer types [48] and in transgenic mouse models of PDAC [28]. Tumor IFP has also been measured in four treatment-naïve PDAC patients, and in that small study, IFP ranged from 6.1 mmHg to 16.6 mmHg [28].

Although MVD, HF_Pim_, and IFP are parameters that are partly determined by the angiogenic activity of the tumor tissue, there were no correlations between these parameters with the only exception that HF_Pim_ decreased with increasing MVD in BxPC-3 tumors. The lack of correlation between HF_Pim_ and MVD in Capan-2 tumors is not unexpected because it has been shown that many vessels in PDACs are occluded and hence nonfunctional due to mechanical pressure from the surrounding connective tissue [20, 21]. The difference between the two PDAC models may be due to a higher fraction of nonfunctional vessels in Capan-2 tumors than in BxPC-3 tumors. Moreover, the observed absence of correlations between IFP and MVD or HF_Pim_ is consistent with most previous studies, which showed a lack of correlation between the IFP of tumors and tumor vascularity or hypoxia, both in patients with cervix cancer [49] and in experimental tumors [48, 50, 51].

Associations between lymph node metastasis and IFP, HF_Pim_, or MVD were searched for by comparing two groups of BxPC-3 tumors, one consisting of tumors giving rise to none or one positive lymph node and the other consisting of tumors giving rise to two or more positive lymph nodes, and three groups of Capan-2 tumors, one consisting of tumors metastasizing to none or one lymph node, one consisting of tumors metastasizing to two lymph nodes, and one consisting of tumors metastasizing to three of more lymph nodes. Ideally, the physicochemical microenvironment of tumors developing metastases should be compared with that of tumors that do not metastasize. However, this comparison was not appropriate because 18 of the 20 Bx-PC-3 tumors and 19 of the 20 Capan-2 tumors metastasized to at least one lymph node. Our splitting of the BxPC-3 tumors resulted in two tumor groups of equal size (10 tumors in each group) and, hence, high power of the statistical analyses. Our division of the Capan-2 tumors resulted in three tumor groups of unequal sizes (4, 12, and 4 tumors), and moreover, by using a splitting equal to that used for the BxPC-3 tumors, two groups of highly unequal sizes (4 and 16 tumors) were compared. Despite suboptimal divisions into tumor groups, IFP was found to be significantly different for the Capan-2 groups as it was for the BxPC-3 groups.

Lymph node metastasis was not associated with MVD or HF_Pim_ in either of the tumor models. The former observation contrasts with clinical studies of many tumor types, which have shown significant correlations between disease-free or overall survival rate and tumor microvascular density [39]. In these clinical studies, MVD was scored by counting vessels in vascular hot spots in the tumor periphery, whereas in our preclinical study, MVD was scored by counting vessels in whole tumor cross-sections. Furthermore, there is significant evidence from clinical and preclinical investigations that extensive tumor hypoxia as well as high angiogenic activity may promote lymph node metastasis [26, 40, 43, 52, 53]. Similar studies of PDAC are sparse; however, there is some evidence from small clinical studies that tumor aggressiveness may be associated with high fraction of hypoxic tissue [33] or highly elevated angiogenesis [18] also in PDAC.

The present study suggests that lymph node metastasis in PDAC may be promoted primarily by interstitial hypertension rather than extensive tumor hypoxia or high angiogenic activity. The BxPC-3 and Capan-2 tumors did not develop intratumoral lymphatics, and our finding is consistent with studies of other tumors without functional intratumoral lymphatics. Thus, studies of melanoma and cervix carcinoma xenografts without intratumoral lymphatics have revealed that lymph node metastasis is associated with high IFP, both in tumors with and tumors without hypoxic regions [41, 42]. However, some patient-derived xenograft models of cervix carcinoma can develop functional intratumoral lymphatics, and functional intratumoral lymphatics may promote lymph node metastasis and reduce tumor IFP, leading to an inverse relationship between IFP and metastatic propensity [54].

Conclusive studies of mechanisms linking high IFP in tumors to lymph node metastasis have not been reported thus far. However, several possible mechanisms have been suggested. High tumor IFP may force interstitial fluid to flow from the tumor tissue into adjacent normal tissues [42], and this fluid flow may direct tumor cells toward peritumoral lymphatics by autologous chemotaxis [55], and may decrease the sentinel lymph node immunity to metastatic tumor cells [56, 57]. Moreover, the interstitial fluid may transport proteolytic enzymes and chemokines that facilitate tumor cell migration by remodeling the extracellular matrix [55], and may carry lymphangiogenic factors that promote metastasis by dilating peritumoral lymphatics and inducing lymphangiogenesis [58].

Tumors develop elevated IFP because they show high resistance to blood flow, low resistance to transcapillary fluid flow, and impaired lymphatic drainage [25, 48]. The resistance to blood flow is determined primarily by the density of the microvascular network and the diameter and tortuosity of the vessels. The transcapillary fluid flow is influenced strongly by the permeability of the vessel walls. The lymphatic drainage is determined by the density of the peritumoral lymphatic network and the tumor-induced dilation of the peritumoral lymphatics [25, 50]. The IFP of tumors is thus expected to show a strong relationship to tumor-induced hem- and lymphangiogenesis.

Several proangiogenic factors associated with interstitial hypertension-associated lymph node metastasis were identified in BxPC-3 and Capan-2 tumors, and three of these factors were up-regulated in high IFP/highly metastatic tumors in both models: TGFB, ANG, and IGF1. High expression of these genes has been shown to be associated with high incidence of lymph node metastases and poor survival rates in several cancer types [59] including PDAC [37, 38, 60]. Up-regulation of TGFB may lead to increased synthesis and cross-linking of extracellular matrix proteins, increased tumor tissue stiffness and solid stress, compression of blood vessels, collapse of intratumoral lymphatics, and highly elevated IFP [61]. Moreover, TGFB protein may promote tumor cell invasion and metastasis by facilitating epithelial-to-mesenchymal transition [62]. ANG stimulates ribosomal RNA (rRNA) transcription, and ANG-mediated rRNA transcription has been revealed to be a general requirement for hem- and lymphangiogenesis induced by other angiogenic factors [63, 64]. Amplified IGF1 signaling has been shown to promote the development and progression of cancer, and furthermore, IGF1 has been identified as an important lymphangiogenic factor in several cancer types [65, 66]. Up-regulation of lymphangiogenic factors like IGF1 may facilitate lymph node metastasis by promoting peritumoral lymphangiogenesis [66, 67].

In addition, high IFP/highly metastatic tumors of the BxPC-3 model showed highly increased expression of VEGF-C, and in this model, the density of peritumoral lymphatics was higher in high IFP/highly metastatic tumors than in low IFP/poorly metastatic tumors. VEGF-C is the most specific and well characterized tumor-derived lymphangiogenic factor identified thus far, and high expression of VEGF-C has been shown to facilitate lymph node and distant metastasis in numerous animal models and many malignant diseases in humans [56, 58, 65, 66]. Our study suggests that lymph node metastasis is promoted by VEGF-C also in PDAC, and furthermore, that high expression of VEGF-C may be associated with high tumor IFP in some PDAC xenograft models. Interestingly, it has been shown that VEGF-A is up-regulated in tumors with highly elevated IFP in some xenograft models of malignant melanoma [68, 69].

Moreover, high IFP/highly metastatic Capan-2 tumors showed increased expression of TNF and members of the CXC chemokine family. TNF may promote tumor-induced lymphangiogenesis by up-regulating the expression of VEGF-C and VEGF-D [70–72], and this cytokine has also been shown to facilitate lymph node metastasis by promoting epithelial-to-mesenchymal transition [73]. The CXCL1, CXCL5, and CXCL6 chemokines have the ELR motif proximal to the CXC sequence, and all ELR containing CXC chemokines have been shown to be potent promoters of tumor hemangiogenesis [74]. CXCL5, which showed by far the highest up-regulation in high IFP/highly metastatic Capan-2 tumors, has been identified as an important angiogenic stimulator in experimental PDAC [75], and furthermore, overexpression of CXCL5 has been shown to be associated with poor survival in PDAC patients [76].

In summary, BxPC-3 and Capan-2 PDAC xenografts developed an abnormal microvasculature during growth, resulting in a physicochemical microenvironment characterized by hypoxia and elevated IFP. Lymph node metastasis was associated with high IFP rather than high MVD or high fraction of hypoxic tissue. Seven angiogenesis-related genes associated with high IFP-associated metastasis were identified in each of the PDAC xenograft models, and three of the genes were common for the two PDAC models. It is possible that the proteins encoded by these genes may have induced high IFP by increasing the microvascular resistance to blood flow, or may have promoted lymph node metastasis by enhancing peritumoral lymphangiogenesis or facilitating epithelial-to-mesenchymal transition. These possibilities merit to be investigated in comprehensive preclinical studies aiming at revealing causal relationships between high IFP and lymph node metastasis, studies that may lead to the identification of novel therapeutic targets for PDAC.

## MATERIALS AND METHODS

### Tumor models

BxPC-3 and Capan-2 (American Type Culture Collection, VA, USA) human PDAC xenografts grown in adult (8-12 weeks of age) female BALB/c *nu*/*nu* mice were used as preclinical tumor models. Tumors were initiated from cells cultured in RPMI-1640 (25 mmol/l HEPES and l-glutamine) medium supplemented with 13% bovine calf serum, 250 mg/l penicillin, and 50 mg/l streptomycin. Approximately 2.5 × 10^6^ cells in 20-30 μl of Hanks’ balanced salt solution were inoculated intramuscularly in the left hind leg, and tumors were included in experiments when having grown to a volume of 400-600 mm^3^, as measured with calipers.

### Metastatic status

Lymph node metastases were searched for by examining six pairs of lymph nodes (Figure 1). The lymph nodes were resected and their sizes were measured with a stereomicroscope before they were fixed in phosphate-buffered 4% paraformaldehyde and prepared for histological examination. Histological sections were cut at 50-μm intervals throughout the entire tissue, stained with hematoxylin and eosin, and examined for metastatic growth by light microscopy. Lungs and liver were prepared in the same way, and histological sections cut at 100-μm intervals were examined for pulmonary and hepatic micrometastases. The peritoneal cavity was examined for macroscopic tumor growth.

### Interstitial fluid pressure

IFP was measured with a Millar SPC 320 catheter equipped with a 2F Mikro-Tip transducer (Millar Instruments, Houston, TX, USA). The catheter was connected to a computer via a Millar TC-510 control unit and a preamplifier, and data acquisition was carried out by using the LabVIEW software. The IFP of experimental tumors is relatively uniform in central tumor regions and drops steeply to normal tissue values at the tumor surface [25, 41, 48], and therefore, IFP was measured in the tumor center in this study. Two measurements were carried out in each tumor by inserting the probe from the lateral side and then from the posterior side, and these two measurements provided highly similar values, consistent with previous studies in our laboratory [77]. Multiple measurements were not conducted because there is a danger that puncture holes may relieve the pressure so that subsequent measurements are not valid.

### Immunohistochemical assessment of tumor hypoxia and vascularity

Histological sections were prepared by standard procedures and stained with hematoxylin and eosin (HE) or immunostained for hypoxia, blood vessels, or lymphatics. Pimonidazole [1-[(2-hydroxy-3-piperidinyl)-propyl]-2-nitroimidazole], injected as described earlier [78], was used as a marker of tumor hypoxia, and CD31 and LYVE-1 were used as markers of blood and lymph vessel endothelial cells, respectively. An anti-pimonidazole rabbit polyclonal antibody (Professor James A. Raleigh, University of North Carolina, Chapel Hill, NC, USA), an anti-mouse CD31 rabbit polyclonal antibody (Abcam, Cambridge, UK), or an anti-mouse LYVE-1 rabbit polyclonal antibody (Abcam) was used as primary antibody. Quantitative studies were carried out on preparations cut through the central regions of tumors, and three sections of each staining were analyzed for each tumor. Blood vessel density was scored by counting CD31-positive vessels in whole tumor cross-sections as described elsewhere [54]. The density of peritumoral lymphatics was assessed by counting LYVE-1-positive vessels in the muscle tissue located within a distance of 0.5 mm from the tumor surface. Fraction of pimonidazole-positive tissue was assessed by image analysis [44] and was defined as the area fraction of the viable tissue showing positive staining.

### Quantitative PCR

The RT^2^ Profiler PCR Array Human Angiogenesis (PAHS-024Z; SABiosciences, Frederick, MD, USA) was used for expression profiling of angiogenesis-related genes. Total RNA was isolated from tumor tissue stabilized in RNA*later* RNA Stabilization Reagent (Qiagen, Hilden, Germany). RNA isolation, cDNA synthesis, and real-time PCR were carried out as described earlier [79]. Fold difference in gene expression was calculated by using the ΔΔC_T_-method [80]. A C_T_-value of 35 (15 cycles above the positive PCR control) was set as detection limit, and hence, all C_T_-values above 35 were set to 35. The arrays included five housekeeping genes [β-actin (ACTB), β-2-microglobulin (B2M), glyceraldehyde-3-phosphate dehydrogenase (GAPDH), hypoxanthine phosphoribosyltransferase 1 (HPRT1), ribosomal protein lateral stalk subunit P0 (RPLP0)], and each C_T_-value of a tumor was normalized to the mean C_T_-value of these genes (ΔC_T_ = C_T_^gene of interest^ - C_T_^mean of housekeeping genes^). Normalized gene expression levels were calculated from three biological replicates as 2^-mean ΔCT^.

### Ethics

The animal experiments were approved by the Institutional Committee on Research Animal Care and were performed in accordance with the Interdisciplinary Principles and Guidelines for the Use of Animals in Research, Marketing, and Education (New York Academy of Sciences, New York, NY, USA) and the EU Directive 2010/63/EU for animal experiments (http://ec.europa.eu/environment/chemicals/lab_animals/legislation_en.htm).

### Statistical analysis

The Pearson product moment correlation test was used to search for correlations between parameters. Curves were fitted to data by linear regression analysis. Comparisons of data were carried out by using the Student *t* test (single comparisons) or by one-way ANOVA followed by the Bonferroni's test (multiple comparisons). The Kolmogorov-Smirnov method and the Levene's method were used to test for normality and equal variance, respectively. Probability values of *P* < 0.05, determined from two-sided tests, were considered significant. The statistical analysis was carried out with SigmaStat statistical software.
